# Modeling for Insights: Does Fiscal Decentralization Impede Ecological Footprint?

**DOI:** 10.3390/ijerph191610146

**Published:** 2022-08-16

**Authors:** Guitao Qiao, Dan Yang, Mahmood Ahmad, Zahoor Ahmed

**Affiliations:** 1 Business School, Shandong University of Technology, Zibo 255000, China; 2 Department of Accounting and Finance, Faculty of Economics and Administrative Sciences, Cyprus International University, Mersin 10, Haspolat 99040, Turkey; 3 Department of Business Administration, Faculty of Management Sciences, ILMA University, Karachi 75190, Pakistan

**Keywords:** fiscal decentralization, technological innovation, economic growth, economic globalization, ecological footprint

## Abstract

In recent years, the debate on environmental issues has become a hot topic. Fiscal decentralization is believed to be a crucial driver of environmental sustainability. However, the discussion on the effect of fiscal decentralization (FD) on environmental sustainability has not reached a unanimous conclusion. In this study, we inspect the effect of fiscal decentralization, economic development, technological innovation, economic globalization, and energy use on environmental quality in eight Asia-Pacific Economic Cooperation (APEC) member countries. In addition, we analyze the mechanisms through which fiscal decentralization influences the ecological footprint (EF) through the channels of technological innovation and economic growth. Using the STIRPAT framework, this study employed the CS-ARDL method for short-run and long-run analyses that deal with slope heterogeneity and cross-sectional dependence. The empirical results show that fiscal decentralization and technological innovation mitigate ecological footprint, while economic development, energy consumption, and urbanization negatively affect environmental quality. However, economic globalization is not related to the EF in the sample economies. The results further reveal that FD enhances environmental quality through the channel of technological innovation, while it does not affect the EF through the channel of economic growth. Finally, it is recommended to make a reasoned division between the rights and responsibilities of local government and central government in environmental pollution management, and optimize the environmental system. At the same time, policymakers should encourage technological innovation to reduce the adverse impacts of economic development and energy consumption on the environment.

## 1. Introduction

According to United Nations Environment Programme (UNEP) [1], the global economy has experienced a fivefold growth over the past 50 years, mainly by using natural resources and energy in production and consumption. At the same time, climate change, loss of biodiversity, and other forms of pollution have reduced environmental quality. Excessive rates of global warming change climate precipitation patterns, and more frequent extreme natural events threaten humanity. The report also calls for accelerating the use of renewable energy, reducing the loss of biological habitat, and taking action to reverse environmental damage before it becomes too late.

Environmental issues, as a topic closely related to human beings, have attracted continuous debate among scholars. The factors affecting the environment and measures to improve environmental sustainability have received widespread attention, and fiscal decentralization is included among such factors. Fiscal decentralization means that local governments will have more autonomy due to the delegation of authority by the central government. The local authorities will have a better insight into the local environmental situation and the demands of the residents; hence, environmental policymaking will be more targeted. Some scholars suggest that competition among local governments will increase environmental protection efforts and raise environmental standards, namely, the phenomenon of “race to the top” [2]. However, there is another opinion regarding the association between FD and environmental issues. Some scholars state that fiscal decentralization will trigger the “race to the bottom” phenomenon where local governments increase pollutant emissions for economic development and short-term gains at the expense of environmental degradation [3].

Asia-Pacific Economic Cooperation (APEC) is the name of a regional economic forum established in 1989. The APEC’s member countries aim to expand prosperity for the people in the region by stimulating innovative, sustainable, and balanced economic growth through regional integration [4]. This study focuses on the APEC economies for several reasons. APEC economies contribute almost 57% to the global GDP and consume almost 60% of the world’s energy. These countries are liable for 70% of global carbon dioxide emissions [5]. APEC countries focus on economic fields, such as trade and investment liberalization and economic and technological cooperation. Although these countries are fiscally decentralized, the current literature neglects the impact of fiscal decentralization on environmental quality in APEC countries. Moreover, insufficient studies have examined the effect of FD on environmental issues using the ecological footprint as a measure of environmental deterioration. From a cutting-edge perspective, while some studies have argued that technological innovation [6,7] and economic growth [8,9,10] are important factors affecting environmental quality, they ignored the combined impact of technology innovation and FD, and FD and economic growth on ecological footprint. 

Based on the above gaps, this paper explores the impact of FD on the EF in a dataset of eight APEC (Australia, Canada, Chile, Japan, Peru, Russia, Thailand, and the United States) countries from 1990 to 2018 and explores the indirect effects of FD on the EF through economic growth and technological innovation channels. Ecological footprint gauges the biologically productive area which is needed for people’s consumption of resources and waste disposal to sustain their lifestyle [11]. The ecological footprint is considered to be more inclusive in determining environmental deterioration than carbon dioxide emissions because it includes not only the carbon footprint component, which is related to CO_2_ emissions, but also five other components, including cropland, forest land, grazing land, built-up land, and fishing grounds [12]. Thus, the pressure of human actions on nature is tracked in terms of six important components. In contrast, CO_2_ emissions are more related to gauging the effect of energy use. EF can measure not only environmental degradation due to human activities [8] but also the extent of sustainable development [13]. Consequently, the ecological footprint is utilized to denote environmental degradation in our paper. Moreover, we applied the CS-ARDL method, which is not only suitable during cross-sectional dependence, heterogeneity of slope, non-stationarity, and endogeneity but also tackles unobserved common factors. 

The main contributions of the present study are as follows. Firstly, the impact of FD on environmental quality in eight APEC countries is examined. Most previous papers in this field are related to the OECD [14], Asian countries [15], and China [3,16], and there is no empirical study in the context of the APEC economies. Hence, this study enriches the relevant literature on environmental sustainability in the Asia Pacific region. Secondly, we investigate the impact of FD on ecological footprint through the channel of economic growth and technological innovation. Exploring this mechanism is helpful to put forward more specific policy suggestions for environmental protection. The existing studies examining FD and the ecological footprint nexus have overlooked the indirect effects of FD on environmental quality through technological innovation and economic growth. Thirdly, we used the ecological footprint to measure environmental sustainability and explore its nexus with FD. Fourthly, we employed advanced econometric estimation methods, which provide results robust to endogeneity, cross-sectional dependence, and slope heterogeneity problems.

The remaining of the present study is structured as below. In Section 2, we review the relevant literature. We provide the theoretical framework, data, and econometric methodology in Section 3. Section 4 reports and explains the empirical results. The conclusions and policy implications are discussed in Section 5.

## 2. Literature Review

### 2.1. Fiscal Decentralization and Environmental Sustainability

Existing opinions on the relationship between FD and environmental sustainability fall into two perspectives, namely linear and nonlinear, and within the linear relationship, studies denote increasing or decreasing effects on environmental sustainability. Some scholars support the theory of ‘race to the bottom’ in the nexus between FD and environmental quality, which argues that local governments have the motivation to formulate loose environmental policies to attract liquid capital for obtaining competitive advantages over other local governments and seeking greater development space [3]. Consequently, the phenomenon of ‘race to the bottom’ reduces the supply of public goods [17], including those related to the environment, and ultimately reduces environmental quality. Moreover, FD may lead to a ‘green paradox’. The imperfect design of environmental policies is likely to backfire and become even more harmful to the environment, despite good intentions [18]. Zhang et al. [16] investigate the impact of FD on CO_2_ emissions in a panel dataset of Chinese provinces. They argue that under Chinese FD, environmental policies significantly increase CO_2_ emissions, causing a green paradox. In addition, Sigman [19] demonstrates that pollution has damaging externalities. Economic competition and the defensive tendencies shown by local governments will increase the possibility of “free-riding” by local governments, thereby increasing pollution emissions and worsening environmental quality. From another perspective, “free-rider” behavior allows different regional governments to benefit from each other’s environmental management achievements for free, thus reducing ecological investment [20]. This facilitation will, up to a certain extent, inhibit the local government’s enthusiasm for environmental protection.

Other scholars suggest that FD boosts environmental quality. Firstly, Tiebout [21] raised the theory of “the vote with the feet”, arguing that residents express their preferences for local public goods in this way; at the same time, local governments have sufficient information on public service demand and pollution status. Thus, the supply of the public good and environmental policies are more responsive to the preferences of local residents [21]. Secondly, FD helps to tailor environmental management [22] and maximizes the public resource allocation efficiency [23] along with eco-energy efficiency [24]. For example, the empirical findings of Zhang et al. [25] show that FD conduces to promoting the prevention and control of industrial waste and haze. Thirdly, under the “beggar-thy-neighbor” effect, FD will trigger the phenomenon of “race to the top” among local governments [2,26], increase the awareness of environmental protection and environmental expenditure [27], and formulate higher environmental quality standards. 

Recently, some empirical and theoretical studies suggest that the link between FD and environmental sustainability may be driven by a non-linear relationship, that is, the inverted U-shape. Phan et al. [15] unfolded the effect of FD on emissions for countries in Asia and found that an increase in revenue and expenditure decentralization significantly reduces CO_2_ emissions, while a decrease in expenditure decentralization raises CO_2_ emissions in the long run. The empirical results confirm that the effect of FD on CO_2_ is asymmetric. The outcomes of Elheddad et al. [28] indicate a non-linear relationship between FD and energy consumption in a sample of Chinese provinces. 

### 2.2. Factors Influencing Environmental Sustainability

The world is experiencing different problems of ecological degradation, for instance, the escalation of energy demand and greenhouse gases. More and more scholars are focusing on factors that affect environmental degradation in order to explore mitigation options for environmental sustainability. Initial studies usually measured environmental degradation in terms of CO_2_ emissions, but this measurement method is weak and inefficient compared with the ecological footprint, which is a comprehensive indicator [29]. The EF is extensively considered as a valid proxy for environmental degradation [30,31]. It is effective in assessing the utilization of ecological resources as well as the impact of human activities on ecology, and it can effectively measure the environmental degradation caused by human activities [8] as well as sustainable development [13].

The existing literature on the influencing factors of environmental quality or environmental sustainability mainly focuses on GDP, natural resources, technological innovation, human capital, urbanization, trade, globalization, and FDI, which could be broadly separated into two groups. On the one hand, environmental sustainability is affected by a series of changes in economic development, energy consumption structure, technological innovation, etc. These changes give rise to human activities, for example, industrialization, urbanization, and modernization within the region. On the other hand, trade, globalization, and foreign direct investment among different regions impact the environment, reflecting the openness to influencing factors. Since the studies on the nexus between some determining factors and ecological footprint are insufficient, this paper inquired into the literature on CO_2_ emission as a supplement.

Economic growth promotes industrialization, increases energy demand, and accelerates the exploitation of natural resources [32]. Production theory indicates that environmental degradation is the direct result of natural resource exploitation and GDP [33]. Over-reliance on non-renewable resources will increase the ecological footprint [34,35]. Economic development will exert great stress on the ecosystem [36]. In addition, many scholars usually explore the nexus of development–environment using the model of EKC, but unfortunately, the results based on different countries and regions are different [8,9,10].

The acceleration of urbanization will increase the demand for public resources such as energy, food, transportation, water resources, land resources, and electricity, which in turn exacerbate energy consumption, generating more pollution [37]. According to statistics, urbanization produces 70% of greenhouse gas (GHG) emissions. Urban expansion affects biodiversity directly and indirectly [38]. 

Previous literature has explored the linkage between technological innovation and EF but did not draw a consistent conclusion. Khattak et al. [39] examined the relationship between technological innovation and CO_2_ emissions in BRICS countries for the period 1980–2016 and showed that innovation activities increase CO_2_ emissions, except in Brazil. Usman and Hammar [40] used a dataset of APEC countries and similarly, the outcomes demonstrated that technological innovation reduces environmental sustainability. However, some scholars consider technological innovation an important factor for environmental sustainability. On the one hand, technological innovation will promote productivity and economic development and develop advanced and clean technologies, which contribute to curbing environmental degradation [6]. On the other hand, technological innovation can improve energy utilization efficiency [41], drive a shift in the energy structure from traditional energy to clean energy [7], promote the development of an efficient energy market [42], and thus reduce the ecological footprint.

The relationship between globalization (as well as trade) and the environment is still controversial [43]. On the one hand, globalization may facilitate the introduction of clean technologies and raise environmental protection awareness, thereby reducing environmental problems [44]. Moreover, the trade component in economic globalization may promote the transformation of the market’s nature and economic structure from the industrial economy to an environmentally sustainable service-based economy and, therefore, improve environmental quality [45]. Contrarily, globalization may cause deterioration of environmental quality through trade [46]. Industries with serious pollution may transfer from countries with high levels of economic development to developing economies [47], and reduce the environmental quality of the new host countries. Arshad Ansari et al. [48] demonstrate that trade and globalization reduce the EF. The nexus between foreign direct investment and EF has not been consistently determined [49].

### 2.3. Literature Gap

The topic of FD and environmental sustainability is subject to a hot debate. Although research in this area is increasing, there are still some gaps. Firstly, in terms of sample selection, few scholars have studied APEC countries. Secondly, existing studies examining FD and environmental issues usually used CO_2_ emissions to measure environmental sustainability or environmental quality, while there is almost no study directly examining the effect of FD on ecological footprint. Finally, most of the previous research works only considered the direct effect of FD on environmental sustainability without exploring the indirect impact through the channels of technological innovation and economic growth.

## 3. Theoretical Framework and Data

### 3.1. Theoretical Framework

At present, there is no consistent conclusion regarding the FD–ecological footprint nexus. The two typical views are ‘race to the bottom’ and ‘race to the top’. The former viewpoint holds that local governments tend to pursue short-term economic benefits and reduce environmental standards to improve economic development [20]. FD may result in a “green paradox”, that the opposite might be achieved [16]. Furthermore, FD increases environmental degradation that may be associated with the existence of the “free-rider” phenomenon [50]. The latter viewpoint argues that FD will trigger the phenomenon of ‘race to the top’ among local authorities [2,26], increase environmental protection awareness and formulate higher environmental standards. Under FD, with the devolution, local governments will have more capacity to allocate resources and improve the efficiency of ecological resource use [23].

Previous studies widely used the IPAT (Integrated Population, Affluence, and Technology) model to examine human activities’ environmental impact. This model is based on very strict assumptions of direct proportionality. Scholars have argued that these assumptions are unrealistic and this rigid model does not allow hypothesis testing. Later, Dietz and Rosa [51] modified this model into the STIRPAT (Stochastic Impacts by Regression on Population, Affluence, and Technology) form to address these limitations. Therefore, the present study relied on the STIRPAT model, which can be written as:

(1) $$I_{it}=aP_{it}^{b}A_{it}^{c}T_{it}^{d}\mu_{it}$$

**Figure 1:**
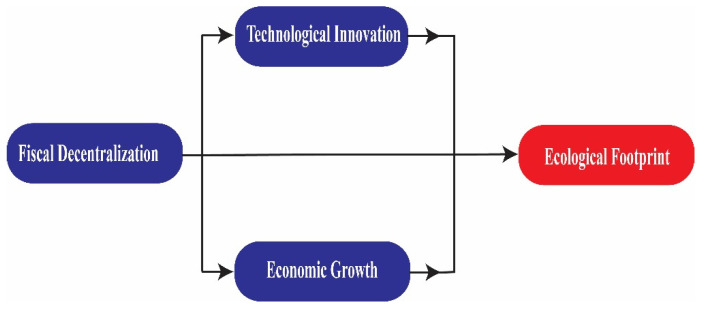
Flow chart for the conceptual framework.

In Equation (1), environmental impact (*I*) is measured by ecological footprint, population (*P*) is represented by urbanization, affluence (*A*) is measured by economic growth (GDP per capita), and technology (*T*) is denoted by technological innovation. In addition, a represents the constant term, *i* denotes cross-sections, and *t* denotes time. The coefficients of population, affluence, and technology are represented by *b*, *c*, and *d*, respectively. This model allows incorporating additional variables that could impact the environment, and thereby, following the studies of Ji et al. [52], and Cheng et al. [53], we expanded the model as:

(2) $$EF=\alpha_{0}+\beta_{1}FD_{it}+\beta_{2}TI_{it}+\beta_{3}GDP_{it}+\beta_{4}EG_{it}+\beta_{5}EC_{it}+\beta_{6}URB_{it}+\varepsilon_{it}$$

where *EF* as a proxy variable for environmental sustainability, denotes ecological footprint; *FD* represents fiscal decentralization; *TI* denotes technological innovation; *GDP* indicates gross domestic product; *EG*, *EC*, and *URB* are control variables that indicate economic globalization, energy consumption, and urbanization, respectively. Adding economic globalization and energy consumption is sensible because these variables influence *EF* and the STIRPAT model is very flexible, unlike the rigid IPAT model. Figure 1 represents the flow chart of our primary model specification.

As mentioned in the literature review, some papers suggest that technological innovation affects ecological footprint [41] and economic growth [10]. In this paper, we perceive that with *FD*, local governments are more proactive in promoting technological innovation with higher authority. Innovative technology can improve energy efficiency and change the energy mix, which in turn can improve environmental sustainability. It seems that technological development is most likely a mediating factor in the process of *FD*’s ecological impact. Therefore, we assume that the nexus between *FD* and ecological footprint will be strengthened with the development of technological innovation. In other words, technological innovation is a determining factor in causing ‘race to the top’. At present, no consistent empirical consensus on the *FD* economic nexus has been reached [54]. Similarly, there is no parallel conclusion about the effects of economic growth on environmental quality, and the concept of EKC is usually introduced to test the relationship between the two [9,55]. Hence, we cannot make an accurate judgment about the role of economic growth between *FD* and ecological footprint linkage. Thus, with the aim of examining the indirect effect of technological innovation and economic growth on ecological footprint, we introduce the interaction terms *FD* × *TI* and *FD* × *GDP* into model-1, as shown in model-2 and model-3, respectively:

(3) $$EF=\alpha_{0}+\beta_{1}FD_{it}+\beta_{2}TI_{it}+\beta_{3}GDP_{it}+\beta_{4}EG_{it}+\beta_{5}EC_{it}+\beta_{6}URB_{it}+\beta_{7}{\left(FD\times TI\right)}_{it}+\varepsilon_{it}$$

(4) $$EF=\alpha_{0}+\beta_{1}FD_{it}+\beta_{2}TI_{it}+\beta_{3}GDP_{it}+\beta_{4}EG_{it}+\beta_{5}EC_{it}+\beta_{6}URB_{it}+\beta_{7}{\left(FD\times GDP\right)}_{it}+\varepsilon_{it}$$

This paper utilizes advanced econometric techniques for empirical assessment, mainly consisting of the seven steps outlined in the Appendix A.

### 3.2. Data

In order to explore the effect of *FD* on *EF*, panel datasets for eight APEC countries from 1990 to 2018 were used. Specifically, the sample countries included Australia, Canada, Chile, Japan, Peru, Russia, Thailand, and the United States. These nations were selected simply because of the availability of the data for empirical analysis. The date of ecological footprint (denoted by *EF*) was obtained from *GFN*, and the date of fiscal decentralization (denoted by *FD*) was obtained from OECD. Furthermore, the datasets on technological innovation (denoted by *TI*) and economic growth (denoted by *GDP*) were obtained from *WDI*. The control variables of economic globalization (denoted by *EG*) and energy consumption (denoted by *EC*) were obtained respectively from SWI and BP, while the data on urbanization (URB) was obtained from the World Bank. Table 1 provides the variable’s description and source. 

## 4. Results and Discussion

In the present research, we applied Breusch and Pagan’s [56] and Pesaran’s [57] methods for cross-sectional dependence checking, and the results are demonstrated in Table 2. As shown in Table 2, all the results indicate the existence of CD. Thus, the sample countries in our dataset are interconnected and dependent on each other. Under the trend of globalization in the modern world, no country will be a lonely island. Various economic activities, ecological changes, cultural spillovers, etc., have integrated all countries. The slope heterogeneity test outcomes are shown in Table 3, which suggests that the model suffers from heterogeneity.

We applied the *CIPS* and *CADF* tests of Pesaran [58] to investigate the integration level of the model parameters. The results are shown in Table 4, where the second and third columns denote the *CIPS* statistics for the level and first-difference forms of the variables, while the fourth to fifth columns represent the *CADF* statistics, respectively. The outcomes demonstrated a mixed order of integration, and all the variables are stationary after taking their first differences.

This study investigated the cointegration links by using the Westerlund panel cointegration test. The findings are provided in Table 5, which shows that the *G_t_* and *P_t_* statistics of all three models significantly reject the null hypothesis. This indicates that the models’ parameters are cointegrated.

This study used the CS-ARDL test to examine the long-run and short-run results for models 1, 2 and 3. This method is more robust than general estimation methods, such as pooled mean group (PMG), FMOLS and DOLS. [59]. CS-ARDL not only deals with cross-sectional dependence, heterogeneous slope, non-stationarity, and endogeneity, but also, more importantly, tackles unobserved common factors. These unobserved common factors can simultaneously affect the ecological footprint, and ignoring these common factors would lead to biased estimation outcomes [60]. The results are shown in Table 6.

The outcomes of the short and long-term showed little difference in coefficient values and significance, as shown in Table 6. FD and technological innovation are negatively related to EF, while GDP, energy consumption, and urbanization are positively related to EF. Further, economic globalization is not significantly correlated with EF. The coefficient of the interaction term of FD and TI shows a negative impact, which is significant at the 10% level, while the interaction term of FD and GDP is not significantly related to EF.

The results in Table 6 indicate that FD significantly decreases the ecological footprint, and on average, the elasticity of FD is −0.049 and −0.072 in the long and short run, respectively. The competition among regions will improve the environmental protection awareness of local governments and set higher environmental standards. Thus, FD enables nations to confront environmental problems with a more positive attitude and build a friendly environment. Under FD, the authority is granted to lower units of the state; thereby, environmental policies will be implemented more effectively and with more specificity, which will improve environmental quality. The outcomes of this paper provide empirical evidence supporting the ‘race to the top’ view.

Technological innovation is negatively related to the ecological footprint as shown in Table 6. More specially, on average, a 0.076% and 0.047% decrease in ecological footprint is caused by a 1% rise in technological innovation in the short and long run, respectively. Technological innovation can bring advanced and clean technologies which contribute to curbing environmental degradation. In addition, innovative improvements in technology, particularly the promotion of renewable energy technologies, can drive the energy transition and reduce pollution. These outcomes resemble the conclusions of Ahmad et al. [7] and Usman and Hammar [40].

A positive and significant nexus is revealed between economic growth and ecological footprint and between energy consumption and ecological footprint, with the average elasticity of 0.303 and 0.269 in the long-run and 0.580 and 0.451 in the short-run, respectively. As economic growth is based on energy consumption, economic development increases the demand for energy, which not only increases the exploitation of natural resources but also increases pollution, thus stimulating ecological degradation. Economic growth may drive urbanization, increase population, etc., which will further reduce ecological carrying capacity. The connection between GDP and EF is analogous to the conclusions of Danish et al. [61] and Udemba [62]. The association between footprint and energy use supports the findings of Charfeddine and Mrabet [9] and Shahzad et al. [63].

In this study, economic globalization is an indicator calculated on the basis of financial and trade globalization, and the findings in Table 6 show that economic globalization is not significantly related to ecological footprint, which is analogous to the finding of Ahmed et al. [45]. There is no empirical evidence for the impact of economic globalization on the ecological footprint in the context of our sample. This estimate also corresponds with the result of Xu et al. [64], who observed no significant nexus between globalization and environmental quality. Moreover, the findings depict that urbanization significantly and positively impacts the EF in the APEC countries. This result can be justified on the ground that urbanization has significantly increased over the past few decades which puts huge pressure on ecological resources. 

The significant negative correlation between the interaction term of FD*TI and the ecological footprint suggests an indirect effect of FD through technological innovation. FD decreases the ecological footprint, and the interaction term of FD and technological innovation further reduces the ecological footprint, showing a channel for FD to improve environmental sustainability. The development of technological innovation has brought new technologies that not only maximize the use of resources but also increase cleaner energy that reduces fossil fuel consumption and ecological footprint. Technological innovation is a significant factor that influences the “race to the top” effect. With the increase in FD, local governments ought to be more environmentally conscious and promote investment in technological innovation to achieve a green environment. Further, the interaction term of FD*GDP is not significantly related to ecological footprint, suggesting that there is no evidence of a mechanism of FD reducing footprint through economic growth in our sample countries. The reason for this result can be the selection of countries with different levels of development. The coefficients of the ECM are −0.82, −0.83, and −0.89 in models 2, 3, and 1, respectively. These coefficients indicate that disequilibrium in the short run is corrected by a high speed of 82%, 83%, and 89% in models 2, 3, and 1, respectively. Thus, the convergence process takes more than one year in every model. These results also indicate that the long-run outcomes can be used for policymaking since short-run deviations are swiftly adjusted. 

With the aim of examining the robustness of the above outcomes, we use the AMG approach and the outcomes are indicated in Table 7. It can be concluded that FD and TI reduce the EF, while GDP and EC increase it. However, EG is not a determinant for explaining the EF. The results are consistent with the outcomes of CS-ARDL. The coefficient of the interaction term (FD*TI) is −0.008, which is significant at the 1% level, indicating that FD has a greater effect on EF when technological innovation is raised. Similarly, the interaction term FD*GDP is not significantly associated with EF, indicating that FD does not impact EF through GDP.

The causal relationships between variables are displayed in Table 8. We noticed one-way causality from FD, technological innovation, and economic globalization to EF, and bidirectional causality between EF and other variables (GDP, EC). There is a bidirectional relationship between FD and TI and between FD and GDP. The findings depict a one-way causality running from urbanization to EF which implies that URB can Granger cause EF but not the other way round.

## 5. Conclusions 

Ecological resources are the premise of human survival and development. The rapid development of modern society is accompanied by the increased utilization and destruction of ecological resources, which have resulted in a series of ecological problems. Using the data from eight APEC countries from 1990 to 2018, we explored the effects of fiscal decentralization (FD), economic development, economic globalization, technological innovation, and energy use on the ecological footprint (EF). Interesting results obtained rom our analysis revealed that FD and technological innovation significantly reduce EF, while GDP, energy consumption, and urbanization increase the ecological footprint. However, no significant impact of globalization on EF was established. 

Secondly, this research tested the indirect influence of FD on EF through GDP and technological innovation by using interaction terms. The outcomes highlighted that FD reduces the ecological footprint through the channel of technological innovation but not through the channel of economic growth. 

## 6. Policy Implications

Based on the results of this paper, several policy implications are offered for promoting environmental sustainability. First of all, imperfections in the structure of the fiscal decentralization system will lead to inefficiency in the supply of public goods, including the environment. Delegating rights to lower levels of government will alleviate environmental degradation to a certain extent; thus, it is vital to make a reasoned division between the rights and responsibility of local government and central government in environmental pollution management. Further, optimizing the fiscal expenditure structure of local governments and increasing their efforts regarding environmental pollution control will increase environmental sustainability. 

Secondly, technological innovation is not only a determinant in reducing the ecological footprint but a path through which FD can indirectly reduce it. The APEC countries should allocate more budget for technological innovation and encourage enterprises to adopt modern technologies that require less energy and generate less pollution. 

Thirdly, although economic growth enhances the quality of life, the contradiction and conflict between environmental quality and economic growth are always the hanging “Sword of Damocles”, If we focus on economic growth without controlling the exploitation and use of non-renewable sources, the survival of mankind may face a bigger crisis in the future. Therefore, economic development and environmental protection should be paid attention to concurrently. 

Fourth, the massive exploitation of fossil fuels will directly cause environmental degradation, and the combustion process of fossil fuels will increase greenhouse gases, exacerbate global warming, and ultimately cause irreversible damage to the environment. Therefore, from the perspective of environmental sustainability, these economies should promote and use green and low-carbon energy (i.e., nuclear and renewables). The harmonious coexistence of resources, environment, economy, and society can be achieved by raising the tax burden on fossil fuel energy, increasing the investment in new energy research and development, and promoting energy conversion.

Finally, the empirical results show that there is a long-term relationship between variables, and policies related to FD, GDP, TI, and EC should be absorbed into the system, in approximately more than one year. When formulating environmental sustainability issues, we should consider the positive effects of fiscal decentralization, economic growth, energy consumption, and technological innovation, and the implementation of policies should be carried out step by step. 

The limitations of this paper are as follows. Firstly, only 8 APEC countries were selected as our sample due to data unavailability for other nations. Secondly, the selection of control variables may be somewhat subjective, and the inclusion of other factors affecting ecological footprint in the model might produce unexpected results. Third, this paper only investigates the indirect effects of FD through technological innovation and economic growth channels. In subsequent studies, some other factors can be considered as the pathways of FD’s effects on environmental sustainability. Moreover, the period of the study is from 1990 to 2018 and some structural breaks can occur during this period due to certain economic or financial crises. However, the methodology used in the study does not allow identifying and incorporating structural breaks in the model. Future studies may incorporate tests for measuring and incorporating structural breaks and financial crises. 

## Figures and Tables

**Table 1:** Variable’s description.

Variable	Symbol	Measurement	Source
Ecological footprint	EF	Global hectares per capita	GFN
Fiscal decentralization	FD	Fiscal decentralization index based on revenue and expenditure decentralization	OECD
Technological innovation	TI	Patent applications (resident + non-resident)	WDI
Economic growth	GDP	Economic growth per capita (constant 2010 $)	WDI
Economic globalization	EG	Index based on financial and trade globalization	SWI
Energy consumption	EC	Energy use (Gigajoule per capita)	BP
Urbanization	URB	Urban population (% of total)	WDI

Note: GFN—Global Footprint Network, OECD—Organization for Economic Co-operation and Development, WDI—World Development Indicators, SWI—Swiss Economic Institute, BP—BP statistical review of world energy.

**Table 2:** CD test results.

Variable	Breusch- Pagan LM	Pesaran Scaled LM	Bias-Corrected Scaled LM
EF	198.058 * [0.000]	22.725 * [0.000]	22.576 * [0.000]
FD	349.468 * [0.000]	42.958 * [0.000]	42.809 * [0.000]
TI	296.577 * [0.000]	35.890 * [0.000]	35.742 * [0.000]
GDP	643.778 * [0.000]	82.286 * [0.000]	82.139 * [0.000]
EG	588.646 * [0.000]	74.919 * [0.000]	74.771 * [0.000]
EC	269.212 * [0.000]	32.233 * [0.000]	32.085 * [0.000]
URB	512.816 [0.000]	64.786 [0.000]	64.638 [0.000]

Note: * < 1%, and [ ] contains the *p*-values.

**Table 3:** Slope heterogeneity test results.

Test	Value	*p*-Value
$\tilde{\Delta }$	8.294 *	0.000
${\tilde{\Delta }}_{adjusted}$	9.816 *	0.000

Note: * < 1%.

**Table 4:** Unit root test results.

Variable	*CIPS*		*CADF*	
	I (0)	I (1)	I (0)	I (1)
EF	−1.682	−5.343 *	−1.428	−3.390 *
FD	−2.646 *	−5.343 *	−2.473 **	−3.585 *
TI	−2.523 **	−5.375 *	−2.119	−3.286 *
GDP	−2.165	−3.052 *	−2.189	−2.743 *
EG	−2.189	−4.914 *	−2.103	−3.314 *
EC	−1.419	−4.518 *	−1.440	−3.152 *
URB	−1.170	−3.493 *	−1.259	−3.819 *

Note: * and ** indicates 1% and 5% level of significance.

**Table 5:** Panel cointegration.

	*G_t_*	*G_a_*	*P_t_*	*P_a_*
Model-1	−3.081 **	−9.508	−8.431 **	−9.789
	[−1.817]	[1.475]	[−2.026]	[0.070]
Model-2	−2.936 **	−10.544	−8.368 *	−9.670
	[−2.050]	[0.435]	[−2.492]	[−0.583]
Model-3	−3.262 *	−11.197	−9.086 *	−10.303
	[−2.956]	[0.194]	[−10.303]	[−0.808]

Note: * and ** indicates 1% and 5% level of significance. [ ] contains the Z-values.

**Table 6:** Results of CS-ARDL.

Variables	Model-1	Model-2	Model-3
Short-run results			
FD	−0.025 * [0.008]	−0.077 * [0.025]	−0.113 * [0.038]
TI	−0.055 ** [0.025]	−0.096 ** [0.041]	−0.078 * [0.044]
EG	0.061 [0.151]	0.134 [0.089]	0.128 [0.104]
GDP	0.684 * [0.198]	0.552 ** [0.226]	0.504 ** [0.213]
EC	0.388 * [0.095]	0.424 * [0.142]	0.542 * [0.193]
URB	0.274 * [0.089]	0.475 * [0.142]	0.474* [0.166]
FD × TI	−	−0.018 * [0.006]	−
FD × GDP	−	−	−0.024 [0.017]
ECM (−1)	−0.899 * [0.078]	−0.819 * [0.096]	−0.834 * [0.097]
Long-run results			
FD	−0.011 * [0.003]	−0.056 ** [0.020]	−0.079 ** [0.035]
TI	−0.031 ** [0.014]	−0.061 ** [0.027]	−0.051 ** [0.023]
EG	0.025 [0.038]	0.073 [0.046]	0.071 [0.058]
GDP	0.356 * [0.104]	0.299 ** [0.128]	0.253 ** [0.124]
EC	0.201 * [0.050]	0.265 * [0.082]	0.341 * [0.135]
URB	0.157 * [0.045]	0.265 * [0.092]	0.556 * [0.206]
FD × TI	−	−0.011 * [0.004]	−
FD × GDP	−	−	−0.015 [0.011]

Note: * < 1%, ** < 5%, and [ ] contains the standard error values.

**Table 7:** Robustness check (AMG).

Variables	Model-1	Model-2	Model-3
FD	−0.073 * [0.027]	−0.092 * [0.024]	−0.101 * [0.031]
TI	−0.032 ** [0.013]	−0.022 ** [0.011]	−0.026 ** [0.011]
EG	0.063 [0.054]	0.070 [0.072]	0.069 [0.071]
GDP	0.918 * [0.254]	0.876 * [0.276]	0.915 * [0.245]
EC	0.301 * [0.109]	0.324 * [0.119]	0.277 * [0.094]
URB	0.473 * [0.167]	0.839 * [0.280]	0.553 * [0.177]
FD × TI	−	−0.008 *** [0.004]	−
FD × GDP	−	−	−0.002 [0.006]
Constant	−10.785 ** [4.432]	−12.191 * [4.975]	−10.982 ** [4.733]

Note: * < 1%, ** < 5%, *** < 10%, and [ ] contains the standard error values.

**Table 8:** The DH non-causality test results.

Variables	EF	FD	TI	GDP	EG	EC	URB
EF	−	6.281 * [4.685] (0.000)	4.707 * [2.874] (0.000)	6.327 * [4.739] (0.000)	5.303 * [3.560] (0.000)	5.441 * [3.719] (0.000)	3.764 * [4.571] (0.000)
FD	5.038 [1.465] (0.142)	−	3.300 *** [1.839] (0.066)	2.765 * [2.863] (0.004)	3.008 * [3.279] (0.001)	2.357 [0.168] (0.866)	3.989 * [4.955] (0.000)
TI	4.235 [1.426] (0.153)	6.452 * [4.883] (0.000)	−	6.040 * [4.408] (0.000)	6.330 * [4.743] (0.000)	3.154 [1.633] (1.087)	2.889 * [3.075] (0.002)
GDP	4.709 * [2.877] (0.004)	3.850 * [4.717] (0.000)	2.201 *** [1.898] (0.056)	−	4.975 * [3.183] (0.001)	6.164 * [4.552] (0.000)	4.481 * [5.796] 0.000)
EG	2.729 [0.596] (0.551)	6.883 * [2.884] (0.004)	1.364 [0.468] (0.639)	5.043 * [3.261] (0.001)	−	3.356 [1.318] (0.187)	1.868 [1.328] (0.183)
EC	3.512 * [4.140] (0.000)	6.708 * [5.178] (0.000)	4.445 ** [2.573] (0.010)	7.241 * [5.791] (0.000)	5.376 * [3.645] (0.000)	−	4.136 * [5.206] (0.000)
URB	1.741 [0.53] (0.589)	6.514 * [4.954] (0.000)	4.040 ** [2.109] (0.034)	4.035 ** [2.101] (0.036)	6.768 * [5.246] (0.000)	1.821 [1.240] (0.214)	−

Note: The symbols *, ** and *** indicate the significance level at 1%, 5%, and 10%, respectively. [ ] and ( ) contain the Z-bar tilde and *p*-values.

## Data Availability

Some or all data and models that support the findings of this study are available from the corresponding author upon reasonable request.

## Appendix A

### Appendix A.1. Econometric Methods

#### Appendix A.1.1. Cross-Sectional Dependence Test

Before estimating the three models we have developed, it is essential for us to inspect cross-sectional dependence (CD). Its presence will cause inconsistent and biased estimation results [52,65] particularly when first-generation methods are preferred for further analysis. In the era of globalization, countries are interdependent and highly correlated, and this will result in high cross-sectional dependence. In this paper, we applied Breusch– Pagan [56] and Pesaran [57] for CD. For the Breusch–Pagan test statistic, the equation is as follows:

(A1) $$CD=T\sum_{i=1}^{N-1}\sum_{j=i+1}^{N}{\hat{\rho }}_{ij}^{2}$$

where ${\hat{\rho }}_{ij}$ is the correlation between errors calculated from Equation (A1), specifically

(A2) $${\hat{\rho }}_{ij}={\hat{\rho }}_{ji}=\frac{\sum_{t=1}^{T}{\hat{\upsilon }}_{it}{\hat{\upsilon }}_{jt}}{{\left(\sum_{t=1}^{T}{\hat{\upsilon }}^{2}{}_{it}\right)}^{1/2}{\left(\sum_{t=1}^{T}{\hat{\upsilon }}^{2}{}_{jt}\right)}^{1/2}}$$

This test is valid for relatively small *N* and sufficiently large *T*, and subsequently, Pesaran [57] proposed the following scaled version alternative:

(A3) $$CD_{LM1}=\sqrt{\frac{1}{N\left(N-1\right)}}\sum_{i=1}^{N-1}\sum_{j=i+1}^{N}(T{\hat{\rho }}_{ij}^{2}-1)$$

However, the above test method is likely to show substantial size distortions when *N* is large and *T* is finite, and the bias is likely to get worse as the number of cross-section units (N) increases [66]. Hence Pesaran [57] proposed a simple alternative that is based on the pair-wise correlation coefficients:

(A4) $$CD_{LM2}=\sqrt{\frac{2T}{N\left(N-1\right)}}\left(\sum_{i=1}^{N-1}\sum_{j=i+1}^{N}{\hat{\rho }}_{ij}\right)$$

#### Appendix A.1.2. Slope Heterogeneity Test

In addition, in this study, we applied Pesaran et al. [67] for the slope heterogeneity test, the equation of the test statistic can be viewed below:

(A5) $$\Delta ={\left(N\right)}^{\frac{1}{2}}{\left(2k\right)}^{-\frac{1}{2}}\left(\frac{1}{N}\tilde{S}-k\right)\;$$

(A6) $$\Delta^{adj}={\left(N\right)}^{\frac{1}{2}}{\left(\frac{2k\left(T-k-1\right)}{T+1}\right)}^{-\frac{1}{2}}\left(\frac{1}{N}\tilde{S}-2k\right)$$

where ∆ and ∆*^adj^* denotes delta title and adjusted delta title. The null hypothesis supports the homogenous slope against the alternative hypothesis.

#### Appendix A.1.3. Unit Root Test

Considering the issue of cross-sectional correlation, we applied Cross-Sectionally Augmented Dickey–Fuller (*CADF*) and Cross-Sectionally Augmented IPS (*CIPS*) for unit root tests by Pesaran [58]. *CADF* and *CIPS* are second-generation unit root tests, and both are robust to cross-sectional dependency and slope heterogeneity.

The empirical form of this test is shown below.

(A7) $$\Delta y_{it}=\alpha_{i}+\beta_{i}{\bar{y}}_{it-1}+\gamma_{i}y_{t-1}+\sum_{j=0}^{k}\delta_{ij}\Delta {\bar{y}}_{it-1}+\sum_{j=0}^{k}\theta_{ij}\Delta y_{it-1}+\varepsilon_{it}$$

where *k* is the lag specification, ${\bar{y\;}}_{t}$ is the temporally defined cross-sectional average. Therefore, we obtain the *CIPS* values utilizing the value of the *CADF* in the above test statistic.

(A8) $$CIPS=N^{-1}\sum_{i=1}^{N}CADF_{i}$$

where $CADF_{i}\;$ denotes the *CADF* test statistic for the ith cross-sectional unit in Equation (A8), and *CIPS* is attained from the mean of *CADF* values for individual cross-sections. The null hypothesis holds up unit roots; in contrast, the alternatives are for no unit roots.

#### Appendix A.1.4. Cointegration Test

In this paper, we used the Westerlund [68] cointegration test to estimate the long-run cointegrating connection between ecological footprint, *FD*, technological innovation, economic growth, economic globalization, and energy consumption. This test is preferred over the panel cointegration test of Pedroni and Kao and can be used when cross-sectional dependency exists in data;, therefore, it is more robust [69]. The test statistics of this test are shown below:

(A9) $$G_{t}=\frac{1}{N}\sum_{i-1}^{N}\frac{{\overset{´}{\alpha }}_{i}}{SE\left({\overset{´}{\alpha }}_{i}\right)}$$

(A10) $$G_{\alpha }=\frac{1}{N}\sum_{i-1}^{N}\frac{T{\overset{´}{\alpha }}_{i}}{{\overset{´}{\alpha }}_{i}\left(1\right)}$$

(A11) $$P_{T}=\frac{\overset{´}{\alpha }}{SE\left(\overset{´}{\alpha }\right)}\;$$

(A12) $$P_{\alpha }=T\overset{´}{\alpha }$$

where Equations (A9) and (A10) represent the group mean statistics, including $G_{t}$ and $G_{\alpha }$. Equations (A11) and (A12) represent the panel statistics, including $P_{T}$ and $P_{\alpha }$. The null hypothesis supports no cointegration, and the alternatives are for cointegration.

#### Appendix A.1.5. Short-Run and Long-Run Estimation

For short and long-run panel estimation, we used the CS-ARDL method proposed by Chudik and Pesaran [70]. This method is more robust than general estimation methods, such as pooled mean group (PMG), FMOLS and DOLS, etc. [59]. CS-ARDL not only deals with cross-sectional dependence, heterogeneous slope, non-stationarity, and endogeneity, but also, more importantly, tackles unobserved common factors. These unobserved common factors can simultaneously affect the ecological footprint, and ignoring these common factors would lead to biased estimation outcomes [60]. Considering the above condition, we estimated model-1 to model-3 with the CS-ARDL approach. The general form of CS-ARDL is given as:

(A13) $$EF_{i,t}=\alpha_{0}+\overset{p}{\underset{j=1}{\sum }}\lambda_{it}EF_{i,t-j}+\overset{p}{\underset{j=0}{\sum }}{\overset{´}{\alpha }}_{it}W_{t-j}+\overset{3}{\underset{j=0}{\sum }}\overset{´}{{\ddot{\upsilon }}_{it}}{\bar{Z}}_{t-j}+\mu_{it}\;$$

where ${\bar{Z}}_{t}$= (${\bar{\Delta EF}}_{t},{\bar{W}}_{t}\prime$)’, and $W_{it}$ denotes a set of explanatory variables, including *FD*, *TI*, *GDP*, *EG*, and *EC*.

#### Appendix A.1.6. Robustness Test

With the aim of testing the robustness of the models, we used the AMG method for testing. AMG not only deals with cross-section dependence [34] but is also useful in the existence of non-stationary variables [65], so the estimation results will be more accurate.

#### Appendix A.1.7. Granger Causality Test

In order to analyze the causal flow, this article employed the Dumitrescu and Hurlin [71] causality test. The DH non-causality test is proposed based on the Granger non-causality test for heterogeneous panel data models. At the same time, this test can handle cross-sectional dependence [52]. The null hypothesis of the method supports no causal link among variables and the alternative hypothesis supports causal relationship. The model of DH non-causality test is expressed as:

(A14) $$z_{i,t}=\alpha_{i}+\overset{p}{\underset{j=1}{\sum }}\beta_{i}^{j}z_{i,t-z}+\overset{p}{\underset{j=1}{\sum }}\gamma_{i}^{j}T_{i,t-j}\;$$

where *j* is for lag length, $\beta^{j}\left(j\right)$ is for autoregressive parameters.
